# Author Correction: Macrophages excite muscle spindles with glutamate to bolster locomotion

**DOI:** 10.1038/s41586-025-09550-6

**Published:** 2025-08-21

**Authors:** Yuyang Yan, Nuria Antolin, Luming Zhou, Luyang Xu, Irene Lisa Vargas, Carlos Daniel Gomez, Guiping Kong, Ilaria Palmisano, Yi Yang, Jessica Chadwick, Franziska Müller, Anthony M. J. Bull, Cristina Lo Celso, Guido Primiano, Serenella Servidei, Jean François Perrier, Carmelo Bellardita, Simone Di Giovanni

**Affiliations:** 1 Faculty of Medicine, Department of Brain Sciences, Imperial College London, London, UK; 2 Faculty of Natural Sciences, Department of Life Sciences, Imperial College London, London, UK; 3 Department of Neuroscience, University of Copenhagen, Copenhagen, Denmark; 4 Faculty of Engineering, Department of Bioengineering, Imperial College London, London, UK; 5 Dipartimento di Neuroscienze, Organi di Senso e Torace, Fondazione Policlinico Universitario Agostino Gemelli IRCCS, Rome, Italy; 6 Dipartimento di Neuroscienze, Università Cattolica del Sacro Cuore, Rome, Italy

**Keywords:** Neuroscience, Neuroimmunology

Correction to: *Nature* 10.1038/s41586-024-08272-5 Published online 4 December 2024

In the version of the article initially published, in Fig. 2j, the 0.5 mm light stimulation trace was inadvertently a duplicate of the 0.5 mm control trace. The figure has now been corrected in the HTML and PDF versions of the article.

