# Supplementary material for: Macrophages excite muscle spindles with glutamate to bolster locomotion
Source: Nature. 2024 Dec 4;637(8046):698–707. doi: 10.1038/s41586-024-08272-5 (PMC11735391; doi:10.1038/s41586-024-08272-5)
Supplement: Supplementary file 1 — Reporting Summary [file 41586_2024_8272_MOESM1_ESM.pdf]

Corresponding author(s): Simone Di Giovanni
Last updated by author(s): Oct 9, 2024

# Reporting Summary

Nature Portfolio wishes to improve the reproducibility of the work that we publish. This form provides structure for consistency and transparency in reporting. For further information on Nature Portfolio policies, see our Editorial Policies and the Editorial Policy Checklist.

## Statistics

For all statistical analyses, confirm that the following items are present in the figure legend, table legend, main text, or Methods section.

| n/a | Confirmed | |
|---|---|---|
| ☐ | ☒ | The exact sample size (*n*) for each experimental group/condition, given as a discrete number and unit of measurement |
| ☐ | ☒ | A statement on whether measurements were taken from distinct samples or whether the same sample was measured repeatedly |
| ☐ | ☒ | The statistical test(s) used AND whether they are one- or two-sided<br>*Only common tests should be described solely by name; describe more complex techniques in the Methods section.* |
| ☒ | ☐ | A description of all covariates tested |
| ☐ | ☒ | A description of any assumptions or corrections, such as tests of normality and adjustment for multiple comparisons |
| ☐ | ☒ | A full description of the statistical parameters including central tendency (e.g. means) or other basic estimates (e.g. regression coefficient) AND variation (e.g. standard deviation) or associated estimates of uncertainty (e.g. confidence intervals) |
| ☐ | ☒ | For null hypothesis testing, the test statistic (e.g. *F*, *t*, *r*) with confidence intervals, effect sizes, degrees of freedom and *P* value noted<br>*Give P values as exact values whenever suitable.* |
| ☒ | ☐ | For Bayesian analysis, information on the choice of priors and Markov chain Monte Carlo settings |
| ☒ | ☐ | For hierarchical and complex designs, identification of the appropriate level for tests and full reporting of outcomes |
| ☐ | ☒ | Estimates of effect sizes (e.g. Cohen's *d*, Pearson's *r*), indicating how they were calculated |

*Our web collection on statistics for biologists contains articles on many of the points above.*

## Software and code

Policy information about availability of computer code

| | |
|---|---|
| Data collection | Data collection, including image acquisition, laser capture microdissection, cell sorting, RNA sequencing, optogenetics, electrophysiological recording, locomotor tests were performed with the specific instrument software installed on the instruments, as detailed in the methods. |
| Data analysis | Image analysis: LAS X (v3.7,4), ImageJ-Fiji (v2.9.0).<br>Flow cytometry: FlowJo (v10.9.0).<br>Electrophysiology: pClamp (v11.2.1)<br>Bulk RNA-seq: bcl2fastq (v2.20), FastQC (v0.11.9), Trim-Galore (v0.6.6), Salmon (v1.6.0), Tximeta (v1.12.4), DESeq2 (v1.34.0), R statistical environment (v4.1.2).<br>scRNA-seq: Cell Ranger Software Suite (v7.1.0), Seurat (v4) in R 4.1.2.<br>Locomotor test: DigiGait™ Imaging system (Mouse Specifics Inc., Boston, MA)<br>Statistic, analysis and graphs: GraphPad Prism (v9.4.0), ggplot2 (v3.3.6), Adobe Illustrator (2022), Adobe photoshop (2024), Microsoft excel, DigiGait™ analysis system (Mouse Specifics Inc., Boston, MA), Matlab 2019b, IBM SPSS Statistics 26.0, online bioinformatics platform (https://www.bioinformatics.com.cn/)<br><br>All RNA sequencing data are available from the NCBI GEO database under accession number GSE244351 and GSE246400. Source data are provided with this paper.<br><br>Code is available at DOI: 10.5281/zenodo.13902719. |

For manuscripts utilizing custom algorithms or software that are central to the research but not yet described in published literature, software must be made available to editors and reviewers. We strongly encourage code deposition in a community repository (e.g. GitHub). See the Nature Portfolio guidelines for submitting code & software for further information.

## Data

Policy information about availability of data

All manuscripts must include a data availability statement. This statement should provide the following information, where applicable:

- Accession codes, unique identifiers, or web links for publicly available datasets
- A description of any restrictions on data availability
- For clinical datasets or third party data, please ensure that the statement adheres to our policy

All RNA sequencing data are available from the NCBI GEO database under accession number GSE244351 (https://www.ncbi.nlm.nih.gov/geo/query/acc.cgi?acc=GSE244351) and GSE246400 (https://www.ncbi.nlm.nih.gov/geo/query/acc.cgi?acc=GSE246400).
Sequences were demultiplexed and adapters trimmed with `bcl2fastq-v2.20 (Illumina). For bulk RNA-seq, read quality controls were carried out using FastQC-v0.11.9 and removing remaining adapters with Trim-Galore (v0.6.6). RNA-seq analysis was run using the COMBINE lab's Salmon (v1.6.0)-DESeq2 (v1.34.0) pipeline in R (v4.1.2). Reads were mapped to the M25 GENCODE reference mouse genome. For scRNA-seq, Samples were de-multiplexed into FASTQ reads and then aligned to the mouse GRCm39 genome reference. Sample de-multiplexing, sequence alignment, barcode processing and single cell 3' unique molecular identifier (UMI) counting were performed by using Cell Ranger Software Suite (v7.1.0) and quality control, data integration and further analysis were performed by using Seurat (v4).

## Research involving human participants, their data, or biological material

Policy information about studies with human participants or human data. See also policy information about sex, gender (identity/presentation), and sexual orientation and race, ethnicity and racism.

| | |
|---|---|
| Reporting on sex and gender | N/A |
| Reporting on race, ethnicity, or other socially relevant groupings | N/A |
| Population characteristics | N/A |
| Recruitment | N/A |
| Ethics oversight | N/A |

Note that full information on the approval of the study protocol must also be provided in the manuscript.

# Field-specific reporting

Please select the one below that is the best fit for your research. If you are not sure, read the appropriate sections before making your selection.

☒ Life sciences ☐ Behavioural & social sciences ☐ Ecological, evolutionary & environmental sciences

For a reference copy of the document with all sections, see nature.com/documents/nr-reporting-summary-flat.pdf

# Life sciences study design

All studies must disclose on these points even when the disclosure is negative.

| | |
|---|---|
| Sample size | The nature of the n is described for each experiment in the corresponding figure legends. Sample size determinations are based on previous experience (Serger et al., 2022) and standards in the field. |
| Data exclusions | No data was excluded |
| Replication | 3-15 independent biological replicates were used for each experiment. The exact number of animals were given in each figure legend for each experiment. |
| Randomization | The biological groups were homogeneous in terms of age (8-12 weeks) and mice. Randomization followed a computerized sequence. Specifically, allocation of mice to control or experimental group was randomised using a random number generator. |
| Blinding | All cell counting and measurements, behavioural tests, and fluorescence analysis were measured in blind. |

# Reporting for specific materials, systems and methods

We require information from authors about some types of materials, experimental systems and methods used in many studies. Here, indicate whether each material, system or method listed is relevant to your study. If you are not sure if a list item applies to your research, read the appropriate section before selecting a response.

## Materials & experimental systems

| n/a | Involved in the study |
|---|---|
| ☐ | ☒ Antibodies |
| ☒ | ☐ Eukaryotic cell lines |
| ☒ | ☐ Palaeontology and archaeology |
| ☐ | ☒ Animals and other organisms |
| ☒ | ☐ Clinical data |
| ☒ | ☐ Dual use research of concern |
| ☒ | ☐ Plants |

## Methods

| n/a | Involved in the study |
|---|---|
| ☒ | ☐ ChIP-seq |
| ☐ | ☒ Flow cytometry |
| ☒ | ☐ MRI-based neuroimaging |

# Antibodies

Antibodies used

Anti-PGP9.5 (Proteintech, 14730-1-AP, 1:500)
Anti-PGP9.5, clone 1C9E11 (Proteintech, 66230-1-Ig, 1:500)
Anti-Tuj1, clone 5G8 (Promega, G7121, 1:500)
Anti-CD45 (R&D, AF114, 1:500)
Anti-F4/80, clone A3-1 (Bio-Rad, MCA497GA, 1:200)
Anti-Iba1, clone EPR16588 (Abcam, AB178846, 1:200)
Anti-CD68, cone PG-M1 (ThermoFisher, MA512507, 1:100)
Anti-B220-FITC, clone: RA3-6B2 (Biolegend, 103228, 1:100)
Anti-Nk1.1, clone: PK136 (Stemcell, 100-0459, 1:200)
Anti-CD3, clone SP7 (Abcam, ab16669, 1:200)
Anti-GFP (Abcam, AB13970, 1:500)
Anti-Collagen IV (Merk, AB769, 1:500)
Anti-Grin2a (Abcam, AB203197, 1:200)
Anti-Gria2 (Merk, AB1768, 1:200)
Alpha-Bungarotoxon Conjugates Alexa Fluor 488 (Invitrogen, B13422, 1:500)
Anti-IL1RA (ThermoFisher, PA5-21776, 1:200)
Anti-HSPA1A (ThermoFisher, PA5-34772, 1:200)
Anti-CD31, clone WM59 (ThermoFisher, MA1-26196, 1:200)
DAPI (Sigma, D5942, 1:1000)
Anti-NeuN-Alexa Fluor488, clone A60 (Merckmilip, MAB377X, 1:100)
Live/Dead-Aqua (Thermofisher, L34966A, 1:200)
Anti-CD45-PE, clone 30-F11 (Biolegend, 103106, 1:100)
Anti-Cd11b-APV/Cyaine7, clone M1/70 (Biolegend, 101226, 1:100)
Anti-F4/80-APC, clone BM8 (Biolegend, 123115, 1:100)
Anti-Cx3cr1-BV605, clone SA011F11 (Biolegend, 149027, 1:100)
Donkey anti-rabbit Alexa Fluor 488 (ThermoFisher Scientific, A21206, 1:500)
Donkey anti-chicken Alexa Fluor 488 (Jackson ImuRes, 703545155, 1:500)
Donkey anti-goat Alexa Fluor 568 (Life Technologies, A11057, 1:500)
Donkey anti-rabbit Alexa Fluor 568 (ThermoFisher Scientific, A10042, 1:500)
Donkey anti-mouse Alexa Fluor 594 (ThermoFisher Scientific, A21203, 1:500)
Donkey anti-rat Alexa Fluor 647 (Abcam, ab150155, 1:200)

Validation

All antibodies used in this study are from commercial suppliers (see notes above for each antibody) that have verified the specificity of the antibodies. All the antibodies have been previously used by various laboratories. All secondary antibodies are verified to not give a specific staining without the primary antibody. Primary antibodies:
Anti-PGP9.5 (Proteintech, 66230-1-Ig 1:500) was validated for immunofluorescent staining in mouse tissue (PMID: 32426489). We further validate this antibody in Figure2.
Anti-PGP9.5 (Proteintech, 14730-1-AP, 1:500) was validated for immunofluorescent staining in mouse tissue (PMID: 38437959). We further validate this antibody in Figure2.
Anti-Tuj1 (Promega, G7121, 1:500) was validated for immunofluorescent staining in mouse tissue (PMID: 34986324). We further validate this antibody in Extended Figure 1.
Anti-CD45 (R&D, AF11, 1:500) was validated for immunofluorescent staining in mouse tissue (PMID: 32221369). We further validate this antibody in Figure2.
F4/80 (Bio-Rad, MCA497GA, 1:200) was validated for immunofluorescent staining in mouse tissue (PMID: 36611965). We further validate this antibody in Figure2.
Anti-Iba1 (Abcam, AB178846, 1:200) was validated for immunoflourescent staining in mouse tissue (PMID: 32863210). We further validate this antibody in Extended Figure 1.
Anti-CD68 (ThermoFisher, Cone PG-M1, MA512507, 1:100). Validated for use in flow cytometry, IHC, IF, WB.
Anti-B220-FITC (Biolegend, clone: RA3-6B2, 103228, 1:200) was validated for immunofluorescent staining in mouse tissue (PMID: 29541074).
Anti-Nk1.1 (Stemcell, clone: PK136, 100-0459, 1:200). Validated for use in flow cytometry, IHC, IF, IP.
Anti-CD3 (Abcam, AB16669, 1:200). Validated for use in flow cytometry, IHC, IF, WB.
Anti-GFP (Abcam, AB13970, 1:500) was validated for immunofluorescent staining in mouse tissue (PMID: 32426489). We further validate this antibody in Figure2.
Collagen IV (Merk, AB769, 1:500) was validated for immunofluorescent staining in mouse tissue (PMID: 35730982). We further validate this antibody in Figure2.
Grin2a (Abcam, AB203197, 1:200). Validated for use in flow cytometry, IHC, ICC/IF, WB. We further validate this antibody in Figure3.
Gria2 (Merk, AB1768, 1:200) was validated for immunofluorescent staining in mouse tissue (PMID: 29941910). We further validate

this antibody in Figure3.
Alpha-Bungarotoxon Conjugates Alexa Fluor 488 (Invitrogen, B13422, 1:500). Validated for use in flow cytometry, IHC, IF. We further validate this antibody in Figure2.
DAPI (Sigma, D5942, 1:1000)
Anti-NeuN Conjugates Alexa Fluor 488 (Merckmilip, MAB377X, 1:100). was validated for immunofluorescent staining in mouse tissue (PMID: 34582785).
Live/Dead-Aqua (Thermofisher, L34966A, 1:200) was validated for flow cytometry. We further validate this antibody in Extended Figure 2.

CD45-PE (Biolegend, 103106, 1:100) was validated for flow cytometry. We further validate this antibody in Extended Figure 2.
Cd11b-APV/Cyaine7 (Biolegend, 101226, 1:100) was validated for flow cytometry. We further validate this antibody in Extended Figure 2.
F4/80-APC (Biolegend, 123115, 1:100) was validated for flow cytometry. We further validate this antibody in Extended Figure 2.
Cx3cr1-BV605 (Biolegend, 149027, 1:100) was validated for flow cytometry. We further validate this antibody in Extended Figure 2.

# Animals and other research organisms

Policy information about studies involving animals; ARRIVE guidelines recommended for reporting animal research, and Sex and Gender in Research

Laboratory animals

Original mouse lines used to generate the mouse lines used in this study:
- C57BL6/J mice: Charles River Laboratories.
- CX3CR1:GFP mice: was gifted by Marzia Malcangio. Details are described in PMID:24743146.
- PvCre : mice from Jakcson Laboratories (known as B6;129P2-Pvalbtm1(cre)Arbr/J), code: 008069
- Cx3Cr1Cre: from the Jackson Laboratories (known as B6J.B6N(Cg)-Cx3cr1tm1.1(cre)Jung/J), code 025524
- R26ChR2: from the Jackson Laboratories (known as B6;129S-Gt(ROSA)26Sortm32(CAG-COP4*H134R/EYFP)Hze/J), code: 012569
- R26NpHR3: from the Jackson Laboratories (known as B6;129S-Gt(ROSA)26Sortm39(CAG-hop/EYFP)Hze/J), code 014539

All male and female mice were used between 8 to 12 weeks of age were used for all experiments. Mice were maintained under standard housing conditions on a 12h light/dark cycle with food and water provided, at a constant room temperature (RT) and humidity (21-24°C and 45-65%, respectively).

Wild animals

No wild animals were used in this study.

Reporting on sex

Both male and female mice ranging from 8 to 12 weeks of age were used for all experiments.

Field-collected samples

No field collected samples were used in this study.

Ethics oversight

Animal work was conducted according to UK Home Office license legislation under the Animals (Scientific Procedures) Act 1986, with Local Ethical Review by the Imperial College London Animal Welfare and Ethical Review Body Standing Committee (AWERB) or, under the EU and Danish legislation to ensure the animals' well-being.

Note that full information on the approval of the study protocol must also be provided in the manuscript.

# Flow Cytometry

## Plots

Confirm that:

☒ The axis labels state the marker and fluorochrome used (e.g. CD4-FITC).

☒ The axis scales are clearly visible. Include numbers along axes only for bottom left plot of group (a 'group' is an analysis of identical markers).

☒ All plots are contour plots with outliers or pseudocolor plots.

☒ A numerical value for number of cells or percentage (with statistics) is provided.

## Methodology

Sample preparation

To sort cells in vivo, mice were anaesthetised with ketamine (80 mg/kg) and xylazine (10 mg/kg). Muscle, heart and lung were dissected and kept in cold DPBS on ice after cardiac perfusion (20 ml DPBS). For muscle macrophages, to reduce the contamination of NMS macrophages, fat, perimysium, nerves and tendons were carefully removed from dissected muscles. Muscle (EDL, soleus, TA and Gastro), heart and lung were transferred to digestion buffers and cut into small cubes. Digestion buffer contained Collagenase B (2 mg/ml), Dispase II (0.83 mg/ml), DNase I (0.25 mg/ml) and DNase I buffer (10 nM Tris Base, 2.5 mM MgCl2, 0.1 mM CaCl2), dissolved in RNase-free DPBS. Tissue suspensions were incubated in 15 ml digestion buffer in a 37 °C water bath for 50 minutes. Tubes were shaken every 5 minutes. 10 ml ice-cold MojoSort buffer (BioLegend) was added to stop digestion. Cell suspension was filtered through 70 µm and 40 µm cell strainers in series. Then, cells were washed with 5 ml cold MojoSort Buffer 2 times and resuspended with 800 µL cold MojoSort buffer for FACS staining. Cell suspension was incubated with mixed antibodies at 4 °C for 20 minutes in the dark. The following antibodies were used: PE-conjugated anti-CD45 (Biolegend, 103106, 1:100), APC/Cyaine7-conjugated anti-CD11b (Biolegend, 101226, 1:100), APC-conjugated anti-F4/80 (Biolegend, 123115, 1:100), Brilliant Violet-conjugated anti-CX3CR1 (Biolegend, 149027, 1:100). LIVE/

DEAD Fixable Aqua Dead Cell Stain Kit (Thermo Fisher Scientific, L34966, 1:200) was used to identify live/dead cells. Stained cells were washed for 2 times with 1 ml cold MojoSort, resuspended with 600 µL MojoSort buffer (with RNase inhibitor, 1:500), filtered through a 40 µm cell strainer and kept on ice until sorting. Becton Dickinson FACS Aria Fusion Flow Cytometer with 100 µm nozzle was set to 4 °C to protect RNA from degradation. Gating was set as FSC-A/SSC-A - Singlet/Doublet - Live/Dead--CD45+-CD11b+-F4/80+-CX3CR1+. Single stain, Fluorescence Minus One (FMO) and negative controls were used for the gating boundaries. Target cells were directly collected into cold collection buffer (DPBS with RNase inhibitor (1:500)).
L4-6 dorsal root ganglia (DRG) from control or macrophage depleted mice were sacrificed, dissected, and snap frozen in liquid nitrogen on day 4. On the day of sorting, DRG were placed into homogenisation buffer (0.25 M sucrose, 25 mM KCl, 5 mM $MgCl_2$, 20 mM tricine-KOH pH7.8, 5 µg/ml actinomycin, 1% BSA, 0.15 mM spermine, 0.5 mM spermidine, EDTA-free protease inhibitor, phosphatase inhibitor, RNase inhibitor). DRGs were homogenised by pelleting with a plastic pestle for 15 seconds. Tritión-X100 (Sigma) was added to the homogenisation mixture to reach the final concentration of 0.1%. Another 15-second stroke was added to further homogenise the DRGs. Finally, DRG homogenisation suspension was filtered through 70 µm followed by 40 µm cell strainers and incubated with NeuN-Alexa Fluor488 antibody (MAB377X Clone A60, 1:100) for 1 hour in cold room with gentle rotation. After washing with washing buffer (homogenisation buffer + 0.1% Triton-X100 + 2% BSA) 3 times, DRG nuclei were resuspended in 2 ml washing buffer and incubated with DAPI for 10 minutes. DAPI+/NeuN+ DRG neuronal nuclei were sorted by Aria III sorter and collected in washing buffer for 10x single nucleus RNA sequencing according to 10X Chromium Next GEM Single Cell 3’ Reagent Kits v3.1 (Dual Index) protocol.

| | |
|---|---|
| Instrument | BD FACS Aria Fusion or BD FACS Aria III |
| Software | FlowJo (v10.9.0) |
| Cell population abundance | Purity of sorted cells or nuclei fraction was above 90%. |
| Gating strategy | Cells were gated as FSC-A/SSC-A - Singlet/Doublet - Live/Dead--CD45+-CD11b+-F4/80+-CX3CR1+ (Extended Figure 2). Neuronal nuclei were gated as FSC-A/SSC-A - Singlet/Doublet - DAPI+- NeuN+ (Extended Data Figure 9). |

☒ Tick this box to confirm that a figure exemplifying the gating strategy is provided in the Supplementary Information.
