# Supplementary material for: Macrophages excite muscle spindles with glutamate to bolster locomotion
Source: Nature. 2024 Dec 4;637(8046):698–707. doi: 10.1038/s41586-024-08272-5 (PMC11735391; doi:10.1038/s41586-024-08272-5)
Supplement: Supplementary file 3 — Supplementary Tables 1–7. [file 41586_2024_8272_MOESM3_ESM.zip › 2023-07-12035G-s3/Supplementary Tables.docx]

**Supplementary Tables**

**Supplementary Table 1. RNA-seq of DE genes after laser capture microdissection.** Shown is the list of DE expressed genes and p-values from dissected MS vs NMS areas.

**Supplementary Table 2. RNA-seq of DE genes from FACS sorted macrophages.** Shown is the list of DE expressed genes and p-values from MSMP, LMP, HMP and SNMP.

**Supplementary Table 3. Single cell RNA-seq dataset from FACS sorted MSMP after optogenetic stimulation of parvalbumin sensory neurons**. Shown is cluster analysis of DE genes.

**Supplementary Table 4. Single nucleus RNA-seq dataset from NeuN positive FACS DRG sensory neurons after macrophage depletion**. Shown is analysis of neuronal subclusters of DE genes.

**Supplementary Table 5. Treadmill locomotion parameters and statistical analysis.**

Shown is a table containing all locomotion parameters analysed and their statistical significance.

**Supplementary Table 6. Treadmill parameters.** Shown is the description of each treadmill parameter.

**Supplementary Table 7. List of antibodies and primers**. Shown is the full list of antibodies and primers used.
